# Prenatal effects of maternal nutritional stress and mental health on the fetal movement profile

**DOI:** 10.1007/s00404-020-05571-w

**Published:** 2020-05-14

**Authors:** N. Reissland, A. R. Millard, R. Wood, B. Ustun, C. McFaul, S. Froggatt, J. Einbeck

**Affiliations:** 1 Department of Psychology, Durham University, South Road, Durham, DH13LE UK; 2 Department of Archaeology, Durham University, South Road, Durham, DH1 3LE UK; 3 Child Study Center, Yale Medical School, New Haven, CT USA; 4 Department of Mathematical Sciences, Durham University, South Road, Durham, DH1 3LE UK

**Keywords:** Fetal movement profile, 4D ultrasound scans, Hyperemesis gravidarum, Catabolism, Maternal stress

## Abstract

**Purpose:**

Prenatal sub-optimal nutrition and exposure to maternal stress, anxiety and depression in pregnancy have been linked to increased postnatal morbidity and mortality. Fetal growth is most vulnerable to maternal dietary deficiencies, such as those evident in hyperemesis gravidarum (HG), early in pregnancy. The purpose of this pilot study was to examine the effects of HG on fetal movement profiles as a measure of fetal healthy development in the 3rd trimester of pregnancy, and to assess whether nutritional stress on the mother can be evaluated using isotopic analysis of hair.

**Method:**

We analyzed fetal movement profiles using 4D ultrasound scans at 32- and 36-weeks' gestation. Fetuses of women (*N* = 6) diagnosed with HG, having lost more than 10% of their body weight in the first trimester of pregnancy were compared to a healthy group (*N* = 6), controlling for stress, depression and anxiety. We tested carbon and nitrogen isotope ratios in maternal hair as a measure of both diet and nutritional changes due to catabolism of body proteins and fats.

**Results:**

HG and catabolism were significantly correlated (*p* = 0.02). Furthermore, at 32-weeks' gestation movement profiles of fetuses of mothers with HG differed significantly from the movement profiles of fetuses of healthy mothers. Fetuses of mothers suffering from HG showed a significantly increased ratio of fine-grained movements at 32 weeks (*p* = 0.008); however, there were no significant differences detectable at 36-weeks' gestation.

**Conclusion:**

The effect of HG on fetal development as expressed by variations in fetal movement profiles in this pilot study suggest that prenatal effects of HG can be measured using movement profiles. Isotope analysis of hair can supplement this with information on nutritional imbalances early in pregnancy.

**Electronic supplementary material:**

The online version of this article (10.1007/s00404-020-05571-w) contains supplementary material, which is available to authorized users.

## Introduction

### Nutritional stress and pregnancy outcomes

Suboptimal nutrition and maternal mental health during pregnancy have been linked to increased infant morbidity and mortality [1, 2]. Although the placenta selectively supplies necessary nutrients throughout pregnancy, fetal growth is most vulnerable to maternal dietary deficiencies (e.g., protein and micronutrients) during placental development early in pregnancy [3]. Not only nutritional status, but also maternal mental health (stress, anxiety and depression) has been identified as a significant prenatal factor predicting postnatal development [4, 5]. The time from conception to 2 years is when individuals are most sensitive to factors leading to chronic disease in adult life [6–8]. Where there is a lack of balanced nutrition, and maternal mental ill-health, it may severely affect fetal neuro-behavioural development [1].

One condition likely to cause nutritional deficiency is Hyperemesis gravidarum (HG). HG is defined by weight loss through excessive vomiting during pregnancy and plays a significant role in health outcomes [9, 10]. These effects are long term, in that in utero exposure to HG is significantly associated with poor postnatal mental health outcomes for the child [10]. Fejzo et al. [10] found a 3.6-fold increased risk of emotional and behavioural disorders of infants born to mothers who suffered from HG. Importantly, there was no effect on adult siblings who were not affected by HG compared with those exposed to HG prenatally. Given that only offspring from mothers who had a poor diet during the pregnancy caused by HG rather than their siblings brought up in the same environment showed negative emotional and behavioural effects, it seems likely that the negative effects are caused by HG and that HG has long-term consequences lasting into adulthood.

### Current study

Although children of mothers with HG have been shown to be affected after birth [3, 6, 7, 11, 12], we know of no research on the health of the fetus. Given that 0.5–3% of pregnant women suffer from diagnosed HG [13, 14], the aim of this pilot study was to test whether HG is reflected in fetal health expressed in fetal movement profiles in later pregnancy, and whether the nutritional stress on the mother can be assessed using isotopic analysis of hair. There is a need for non-invasive measures to test effects of HG during pregnancy with the potential to establish not only growth parameters, but also cognitive and affective parameters [5, 15]. With the advent of 4D ultrasound scanning, it is now possible to establish fetal movement profiles as a way of assessing their development. Such techniques have the potential for deriving diagnostic tools aimed at the early non-invasive detection of developmental dysfunction [15–19].

Although the effects of nutritional stress and mental stress have been reported in separate studies, their relative effects have not been tested. One way in which we can assess nutritional status is to use hair isotope analysis, yielding longitudinal information, from preconception through to the time of sampling at 36-weeks’ gestation. Mental health measures, including stress, anxiety and depression can be assessed by standardized questionnaires. By combining both types of tests with the longitudinal data from 4D scans at 32- and 36-weeks’ gestation, this study investigates for the first time both the effects of maternal nutritional status and maternal stress, anxiety and depression on the development of the fetus.

In this feasibility study, we examined fetal movement profiles of two groups of mothers, six mothers who had suffered HG and six healthy mothers, using 4D ultrasound scans recorded at 32- and 36-weeks’ gestation. We tested for the presence of, and recovery from, under-nutrition catabolism in early pregnancy using maternal hair that grew from before conception to 36-weeks’ gestation by comparing maternal carbon isotope ratios (expressed as δ^13^C) and nitrogen isotope ratios (δ^15^N) in their hair reflecting dietary inputs.

We hypothesized first that HG is significantly associated with catabolism as evidenced by hair samples and second that HG in the first trimester of pregnancy affects the fetal movement profile independent of maternal stress, depression and anxiety.

### Stable isotopes in hair

The ratios of the stable isotopes of carbon (^13^C/^12^C) and nitrogen (^15^N/^14^N) vary in humans due to external variations in food sources and internal metabolic processes. By convention, the ratios are expressed as fractional deviations from the ratios of standard materials, with the notation δ^13^C and δ^15^N, and given as parts per thousand (per mille, ‰) [20]. The major sources of external variations for δ^13^C are differences in photosynthesis, with three classes of food relevant for humans. The majority of plants, and the animals that consume them, use the C_3_ photosynthetic process and produce foods with a mean δ^13^C of − 25‰. Some tropical grasses, including maize and sugarcane, use the C_4_ photosynthetic process and produce foods with a mean δ^13^C of − 13‰. In marine ecosystems, the dissolution of carbon dioxide into seawater decreases the δ^13^C to a mean of − 17‰. Variations in δ^15^N are mainly driven by changes at each step in the food chain, with δ^15^N values increasing by 3–5‰ for each step in the food chain, starting with atmospheric nitrogen at 0‰, and increasing through plants, herbivores, omnivores, carnivores and super-carnivores. Food chains in marine and freshwater ecosystems tend to be longer than in terrestrial ecosystems so that fish can have higher δ^15^N than terrestrial animals.

At homeostasis with a consistent diet, protein in a consumer body has δ^15^N and δ^13^C higher than diet by 3–5‰ and 0–1‰, respectively, while body fat has a lower δ^13^C than body proteins by about 5‰ [21]. During growth, (including weight gain in pregnancy) positive nutrient balance is predicted to slightly lower both δ^13^C and δ^15^N in newly formed tissues, as intake of foods with lower ratios than the body exceeds output of waste. During weight loss, catabolism of the body’s own tissues occurs, drawing on protein for nitrogen and partly on fats for carbon. New tissues formed during weight loss are thus expected to have raised δ^15^N and lowered δ^13^C compared to those formed under homeostasis. The changes in δ^13^C and δ^15^N during catabolism and recovery, when plotted against time, typically show opposing covariance [22, 23], with diverging and then converging values, a feature informally termed ‘bubble’.

These predictions have been borne out in small-scale studies of hair isotopes in humans. A study of ten pregnant women showed δ^15^N correlated with weight gain but detected no change in δ^13^C [8]. However, δ^15^N was shown to increase during HG in a study of eight pregnant women [8], also with no detectable change in δ^13^C (their Table 2 shows mean change + 0.1 ± 0.1 ‰). Two studies of recovering anorexics, with six [22] and seven [23] participants, showed declining δ^15^N and increasing δ^13^C as body mass increased. After the initial changes, some of these individuals showed other patterns due to significant changes in diet, such as vegetarian to omnivorous. Another study of 13 deceased individuals who suffered a period of severe starvation shortly prior to death showed decreasing δ^13^C and increasing δ^15^N [24]. Assuming no isotopic change in the diet, the expected isotopic changes in hair during a pregnancy with normal weight gain are therefore declines in both δ^15^N and δ^13^C, but, in contrast, during HG hair is expected to show increasing δ^15^N and declining δ^13^C. With recovery from HG, δ^15^N should decrease and δ^13^C increase in hair, followed by both decreasing when the pregnancy returns to normal. The changes in δ^13^C, however, may be small or close to zero, and may also be hard to detect in the presence of other factors such as measurement uncertainty and variations in diet.

## Methods

### Participants

Twelve women, whose fetuses were determined healthy at their 20-week anomaly scan, and who had a healthy pre-pregnancy BMI, were recruited through opportunity sampling. Two groups of pregnant women were recruited: six women having a healthy pregnancy with normal weight gain and six women who were diagnosed with HG by a medical practitioner and had lost at least 10% of their body weight in the first trimester of pregnancy (see Tables 1 and 2). However, because the recruitment protocol required a healthy 20-week anomaly scan, we did not record weight loss data in the first trimester. All participants were white British from the North East of England. All women underwent 4D ultrasound scanning at the Windows to the Womb Clinic, Gateshead, UK by experienced sonographers, lasting around 15–20 min at 32- and 36-weeks' gestation. Fetal fine-grained mouth movements were coded frame-by-frame using the Fetal Observable Movement System (FOMS) [15, 16]. The scanning took place with mothers lying in a darkened room on their back or on their side, depending on the position of the fetus in the womb and on the mother’s comfort. The fetal face and upper torso were visualized by means of 4D full frontal and facial profile ultrasound recordings, as well as traditional 2D images. The scans were recorded for off-line analysis with a GE Voluson E8 or E10 Expert Ultrasound System using a RAB6-RS transducer. Mothers received a DVD copy of their scans. Mothers completed the Perceived Stress Scale (PSS) questionnaire, assessing stress levels at each scan [25, 26]. The PSS is a widely used valid and reliable ten-item five-point Likert-based scale measuring the degree to which mothers perceive their life as stressful (ranging from 0 = ‘no stress’ experienced during the last month to 4 = ‘very often’ stressed). Additionally, mothers completed the Hospital Anxiety and Depression Scale (HADS; with two sub-scales HADS-A refers to anxiety and HADS-D refers to depression) [27]. The scale has 14 items [seven items for anxiety and seven for depression and ranges from 0 (minimum) to 21 (maximum)]. Mothers also completed the Pregnancy-Unique Quantification of Emesis/Nausea (PUQE) index resulting in mild (score ≤ 6), moderate (score = 7–12), and severe (score ≥ 13) [28] and an attachment scale [29].

**Table 1:** Participant variables (*N* indicates the number of participants for whom we could obtain this information)

	Healthy group	HG group
Maternal age	*N*(6) *M* = 24.17 SD-5.04	*N*(6) *M* = 26.67 SD = 4.18
Level of education	3 to GCSE level 2 to College/A-level 1 to Bachelor’s degree level	1 to GCSE level 3 to College/A-level 1 to Bachelor’s degree level 1 to Master’s degree level
Fetal head circumference at 20 weeks	*N*(5) *M* = 16.72 SD = 9.58	*N*(6) *M* = 17.43 SD = 4.64
Birth weight	*N*(6) *M* = 3483 SD = 750.26	*N*(6) *M* = 3451.50 SD = 282.03
Apgar score 1 min	*N*(5) *M* = 8.80 SD = .45	*N*(4) *M* = 8.25 SD = 1.5
Apgar score 5 min	(*N* = 5) *M* = 9 SD = .0	*N*(4) *M* = 9 SD = .82
Fetus sex	Two boys, four girls	Three boys, three girls

**Table 2:** Observed variables

Variable	Healthy sample 32 weeks	Healthy sample 36 weeks	HG sample 32 weeks	HG sample 36 weeks
HADS anxiety	*N* = 6 *M* = 3.67 SD = 1.86	*N* = 6 *M* = 3 SD = 3.11	*N* = 6 *M* = 11.17 SD = 5.74	*N* = 6 *M* = 10.67 SD = 6.38
HADS depression	*N* = 6 *M* = 2.67 SD = 1.86	*N* = 6 *M* = 3.17 SD = 2.64	*N* = 6 *M* = 11.17 SD = 2.23	*N* = 6 *M* = 11.17 SD = 3.49
Total maternal attachment	*N* = 6 *M* = 83.67 SD = 8.55	*N* = 6 *M* = 85.83 SD = 3.92	*N* = 6 *M* = 82.83 SD = 6.37	*N* = 6 *M* = 83.00 SD = 8.74
PUQE	*N* = 6 *M* = 3.17 SD = .41	*N* = 6 *M* = 3.67 SD = 1.21	*N* = 6 *M* = 8.17 SD = 3.37	*N* = 6 *M* = 6.83 SD = 4.31
PSS	*N* = 6 *M* = 9.33 SD = 5.61	*N* = 6 *M* = 9.67 SD = 5.13	*N* = 6 *M* = 23.00 SD = 4.78	*N* = 6 *M* = 20 SD = 7.87
Mean fetal exact age at scan	*N* = 6 *M* = 227 (days) SD = 2.630	*N* = 6 *M* = 255 (days) SD = 2.141	*N* = 6 *M* = 228 (days) SD = 4.85	*N* = 6 *M* = 256 (days) SD = 2.63

### Hair sample analysis

Nutritional status was tested with hair, at least 10 cm in length collected at 36-weeks' gestation in both the HG and heathy groups of women. Human hair grows at an average of 1 cm per month [30, 31] therefore collection of 10 cm long hair at 36 weeks allows month by month analysis of carbon and nitrogen isotopes from preconception through to 36-weeks' gestation. A group of ten or more hairs were bound with adhesive tape to keep them aligned before cutting as close to the scalp as possible. Hair samples were coded with anonymous codes. Each bundle of hair was cut into 1 cm segments, corresponding approximately to growth from 2 months prior to conception up to 8 months of gestation. The 1 cm segments were placed in individual micro tubes, and prepared using a modification of previous protocols [22]. To remove fats from the hair, samples were washed three times by ultrasonication in 1 ml of 2:1 methanol: chloroform. The solvent was removed by ultrasonication in 1 ml of deionised water and the samples dried at 55 °C.

Samples were analysed by IsoAnalytical Ltd (Crewe, Cheshire). After weighing into tin capsules, samples were combusted on a Europa Scientific elemental analyser, and isotope ratios measured on a Europa Scientific 20–20 Isotope Ratio Mass Spectrometer. The reference material was IA-R068 (soy protein, δ^13^C_VPDB_ = − 25.22 ‰, δ^15^N_AIR_ = 0.99 ‰), and quality control check samples were IA-R068, IA-R038 (L-alanine, δ^13^C_VPDB_ = − 24.99 ‰, δ^15^N_AIR_ = − 0.65 ‰), IA-R069 (tuna protein, δ^13^C_VPDB_ = − 18.88 ‰, δ^15^N_AIR_ = 11.60 ‰) and a mixture of IAEA-C7 (oxalic acid, δ^13^C_VPDB_ = − 14.48 ‰) and IA-R046 (ammonium sulfate, δ^15^N_AIR_ = 22.04 ‰), which are calibrated against the international standards IAEA-CH-6 and IAEA-N-1. Every fifth sample was measured in duplicate. The technical error of measurement from the duplicate samples was 0.06 ‰ for δ^13^C and 0.05 ‰ for δ^15^N.

Using conventional methods, catabolic and non-catabolic isotope profiles were classified by eye. The anonymized data series for each woman was classified by one of the authors (Millard), without knowledge of their HG status, as showing evidence for catabolism, where there was a clear opposing covariance in the data, possible evidence for catabolism, where there was less clear evidence for opposing covariance, or no evidence for catabolism, where there was no opposing covariance.

### Statistical analysis

The data consist of repeated measures, with each fetus scanned for 15–20 min at 32- and 36-weeks' gestation and fetal fine-grained movements coded frame by frame. The total number of movements serves as dependent variable, which is related through a log-linear model to explanatory variables including stress, depression and anxiety scores as well as effects of gestational week and HG. Further predictors including maternal age, fetal age and education level were initially considered (see supplementary information S3) but removed from the analyses, which follow, as they did not carry significant effects. Since the observed codable scan length varied between fetuses, the model further includes an offset of log codable scan length, thus, effectively modelling total movement rate. Finally, there is a random effect term assessing between fetus variability. The model can hence be formulated as (*H*):

$$\begin{aligned} \log \lambda_{{{\text{it}}}} & = \log \left( {{\text{scan}}\;{\text{length}}_{{{\text{it}}}} } \right) + \beta_{0} \beta_{1} \;{\text{gestational}}\;{\text{age}}_{{{\text{it}}}} \\ & \quad + \beta_{2} {\text{HG}}_{i} + \beta_{3} \times {\text{HG}}_{i} \;{\text{gestational}}\;{\text{age}}_{{{\text{it}}}} \\ & \quad + \beta_{4} {\text{PSS}}_{{{\text{it}}}} + \beta_{5} {\text{HADS}}\_A_{{{\text{it}}}} + \beta_{6} {\text{HADS}}\_D_{{{\text{it}}}} + u_{i} \\ \end{aligned}$$

where the total number of movements ${C}_{it}$ of fetus $i=1,\dots ,12$ at time $t=32, 36$ follows a negative Binomial distribution

$$C_{it} \sim {\text{NB}}(\lambda_{it} ,\theta )$$

and the random effect a Gaussian distribution

$$u_{i} \sim {\text{Normal}}(0,\sigma_{u}^{2} )$$

The three predictor variables in the final row of (*H*) are continuous variables, while HG is a two-level-factor variable indicating the health status, that is the absence (= 0) or presence (= 1) of HG. The variable GestationalAge could be described by either a continuous variable or a two-level factor for weeks 32 or 36. For ease of interpretation, we work with the latter in this paper, with week 32 as reference category. The conclusions do not change if the continuous version is used. We will also, later, consider a similar model (*C*) in which HG is replaced by the two-level factor catabolism (with one level for “yes and possible” and one level for “no catabolism”).

We have opted here for a Negative Binomial response model rather than Poisson as in comparable studies [16, 32, 33], since over dispersion for these data was relatively strong. The $\theta$ parameter takes values of 4.25 and 3.44 in models (*H*) and (*C*), respectively, where $\theta \to \infty$ would correspond to “no over dispersion”. The corresponding likelihood ratio (LR) tests favour the Negative Binomial model with *p* values < 0.0001 in each case. Hence, using the Poisson response for these data would lead to incorrect standard errors with overstated significances. We fitted the model using the glmmTMB function in the glmmTMB library in R [34]. The significance of model parameters was assessed by Wald test *p* values on the parameter coefficients, with *p* values less than 0.05 considered significant.

## Results

All 24 movement scans were successful typically yielding 8–12 min of codable scan allowing rates to be determined. Variations in length of coding were statistically controlled. Reliability testing was conducted on 16% of the sample for which the mean Cohen's Kappa was 0.93 with a range of 0.83–0.98.

**Fig. 1:**
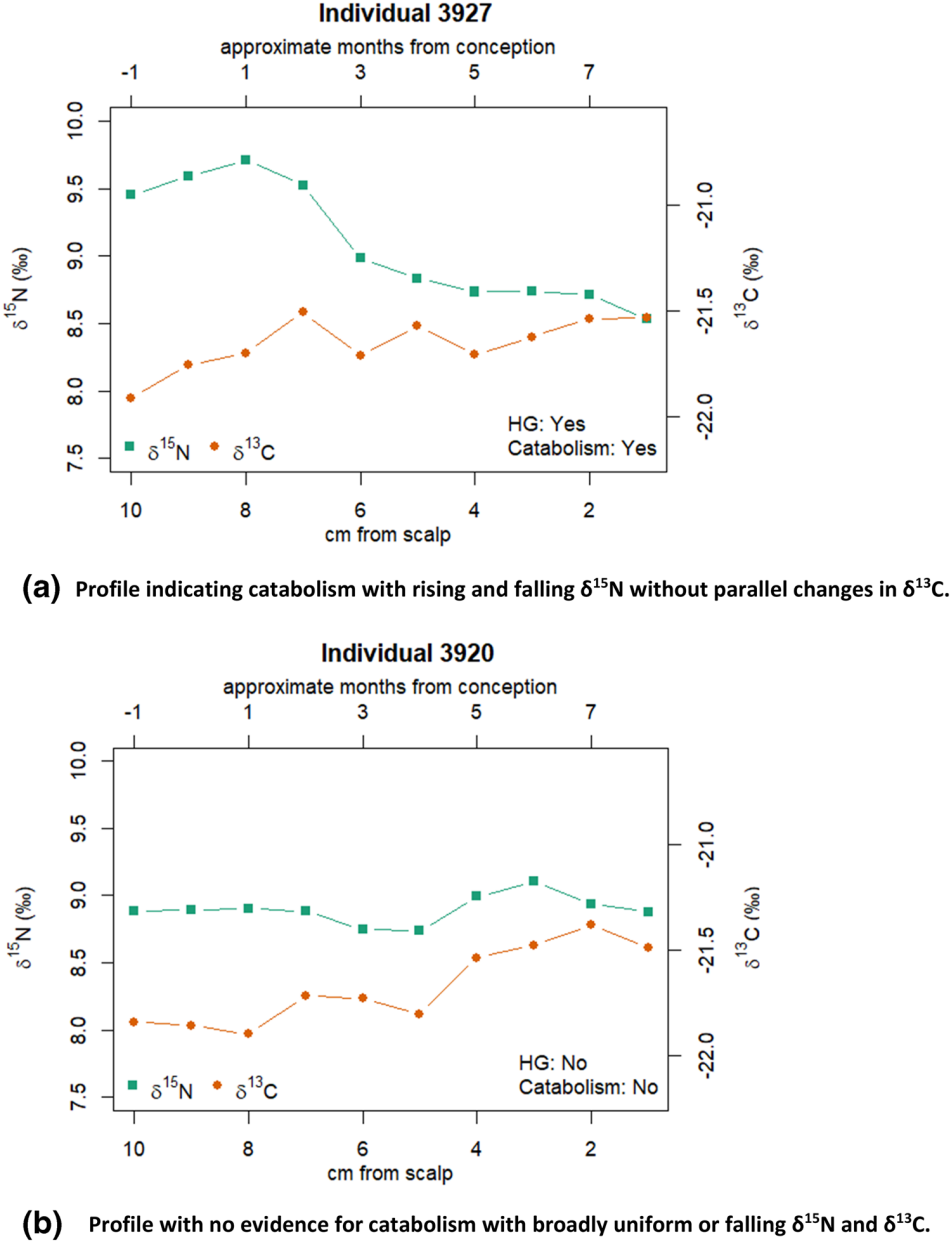
Examples of isotope profiles from hair. **a** Profile indicating catabolism with rising and falling δ^15^N without parallel changes in δ^13^C. **b** Profile with no evidence for catabolism with broadly uniform or falling δ^15^N and δ^13^C

Isotope profiles were obtained for all women (supplementary information S2). One subsample (3935–07) was rejected as showing a *C*:*N* ratio too high for hair. Figure 1 shows examples of catabolic and non-catabolic profiles, and a full set of profiles is in supplementary information S1, together with their HG status and catabolism classification.

### Independent variable analysis

Before modelling the fetal movement profiles, we conducted an explanatory analysis on the maternal factors: HG status, presence of catabolism, stress, depression and anxiety. There were strong interactions between these independent variables which were taken account of in the analysis. Despite these correlations, the variables may still have differential effects on the total number of movements.

#### HG and catabolism

The contingency table of catabolism and health status (Table 3) indicates an association between HG status and the presence of catabolism, which is confirmed by an unconditional exact test for 2 × 2 contingency tables, with *p* = 0.020 [35]. There was an entire absence of catabolism for healthy mothers, which further strengthens the link between HG and catabolism.

**Table 3:** Contingency table of health status and catabolism frequencies

Catabolism	Health status	
	Healthy	Hyperemesis
No	6	2
Yes/possible	0	4

Reported frequencies refer to individual fetuses; there are hence 2 × 12 = 24 total scans coded frame by frame and measurements reported

#### Nutritional stress and mental health

Scores for stress, anxiety, and depression, are significantly related to health status (t-tests, each *p* < 0.001) and two scores to catabolism (PSS: *p* = 0.032, HADS_D: *p* = 0.0015), but not to gestational week (each *p* > 0.7). The three scores are highly correlated with each other, with all correlation coefficients between 0.82 and 0.90.

### Fetal movement analysis

Table 4 shows estimates (non-italic rows), standard errors and p values of the fitted model (*H*). All included parameters except PSS (stress) and HADS_A (anxiety) are significant at the 5% level. From the formulation of the model, it follows that the coefficients have an exponential and multiplicative impact on the movement per scan length rate. Several aspects of the model deserve further discussion.

**Table 4:** Coefficient estimates of model (*H*)

Coefficient	Estimate	SE	*z* value	*p* value
Intercept	− 3.8996 − *3.7136*	0.3160 *0.2840*	− 12.340 − *13.077*	< 2e−16 < *2e*−*16*
HG	1.4507 *1.0626*	0.5470 *0.3896*	2.652 *2.727*	0.0080 *0.0064*
GestationalAge	0.7174 *0.6364*	0.3413 *0.3214*	2.102 *1.980*	0.036 *0.047*
HG:GestationalAge	− 1.1946 − *1.3910*	0.4807 *0.4440*	− 2.485 − *3.133*	0.013 *0.0017*
PSS	0.0645	0.0384	1.679	0.093
HADS_A	0.0463	0.0432	1.072	0.284
HADS_D	− 0.1999	0.0760	− 2.631	0.0085

Italic rows refer to the model fitted without stress, depression and anxiety

#### HG effects

**Fig. 2:**
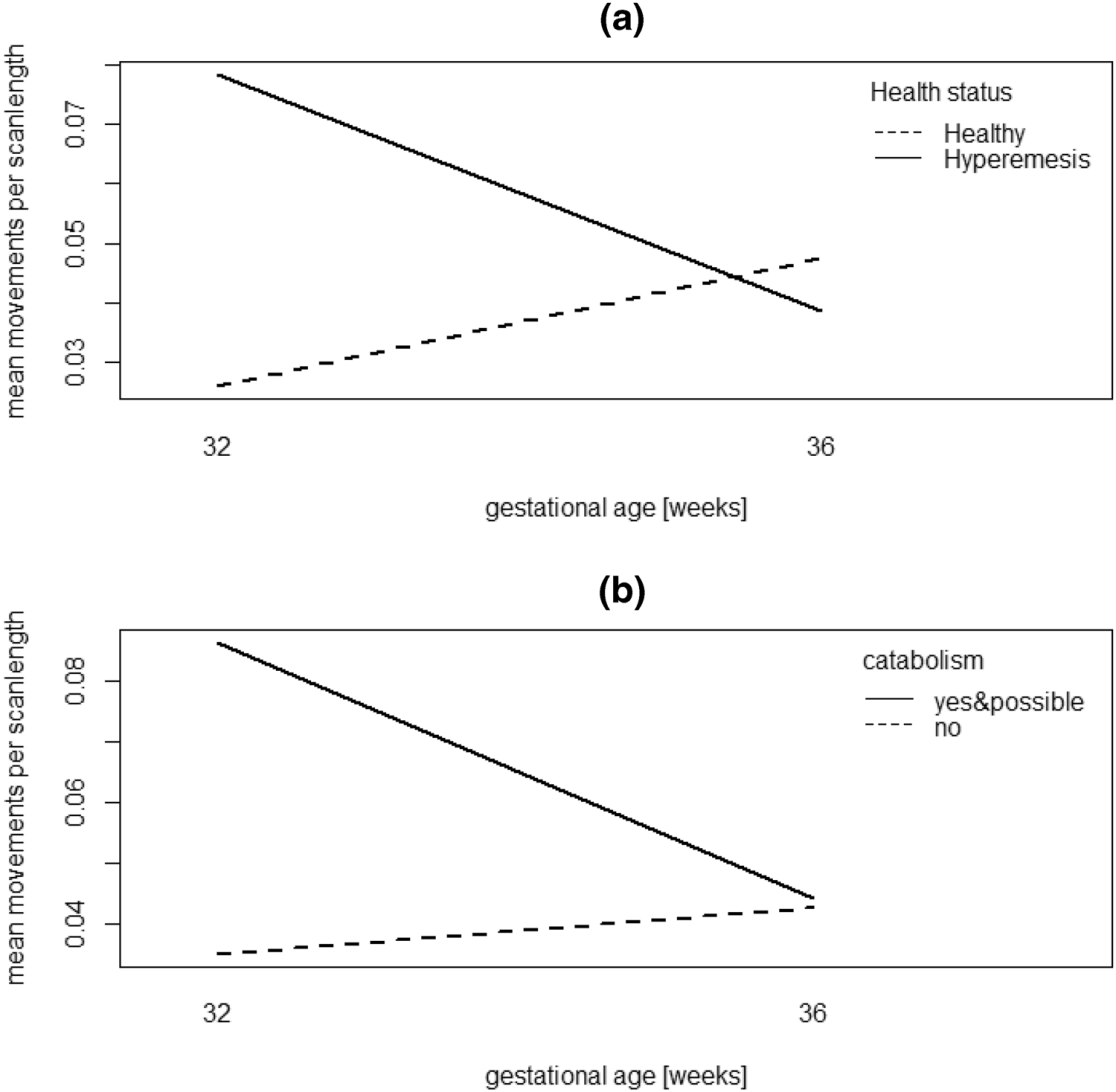
Interaction plots. **a** Interaction plot for health status and gestational age; **b** interaction plot for catabolism and gestational age. Line endpoints correspond to means of the `total number of movements divided by scan length', for the respective factor level combinations. The scan length is measured in seconds [s], so the vertical axis has the unit 1/s

We see that at week 32, after adjusting for stress, anxiety, and depression, the movement rate is larger by a factor of exp(1.4507) = 4.27 for the HG group compared with the healthy group. From Table 4, we know that this a significant effect (*p* = 0.008, Cohen’s *d* = 1.34), which is also clearly visible by the large gap in the left-hand side in Fig. 2a. In contrast, at week 36, the difference between healthy and HG groups is not significant (*p* = 0.577, Cohen’s *d* = − 0.14). Also, for fetuses from the healthy group, from exp(0.717) = 2.05 one infers a two-fold increase in movement rate from week 32 to week 36 (this is the dashed line in Fig. 2a). However, if fetuses are from the HG group, the change from week 32 to week 36 is of magnitude exp(0.717) × exp(− 1.195) = 0.620, that is a reduction by 38%, and this difference (between two-fold increase and a 38% decrease) in the effect of gestational week for the two groups corresponds to the significant (*p* = 0.013) interaction term in Table 4.

#### Catabolism effects

If we replace the factor for HG in the analysis by catabolism, we would expect from previous considerations a similar picture. However, given the small sample size, the catabolism effect seems less pronounced than the reported HG diagnosis with the loss of 10% of body weight in the first trimester (see Fig. 2b and supplementary information S4). The variables concerning catabolism and gestational group do not achieve significance either individually or jointly (p value of LR test 0.426 at 3 degrees of freedom [*df*]). This changes only to a limited extent when not including the score variables (the *p* value of a LR test for joint inclusion of catabolism, gestational age, and their interaction, is now at 0.185 at 3 *df*; with the *p* value for catabolism by itself at *p* = 0.025). We conclude that, albeit strongly associated with HG, catabolism as identified by hair samples seems to be underpowered and in this pilot study is overall a less useful predictor for fetal movements.

#### Maternal mental health

From the model output and the previously described collinearity of the score variables (HADS_A for anxiety, HADS_D for depression, and PSS for stress), we see that, not all these variables appear relevant to the model. Removing PSS and HADS_A from the model renders however HADS_D non-significant (not shown), and a likelihood ratio test for the joint inclusion of the three score variables against their exclusion gives moderate evidence towards their inclusion (*p* = 0.098 at 3 *df*). However, importantly, the main reason for inclusion of the score variables is to evaluate nutritional impact on fetal movements over and above the effects of anxiety, depression and stress. The effect size of the score variables for the model fitted in Table 4 is *η*^2^ = 0.472.

### Distinct movement analysis

**Fig. 3:**
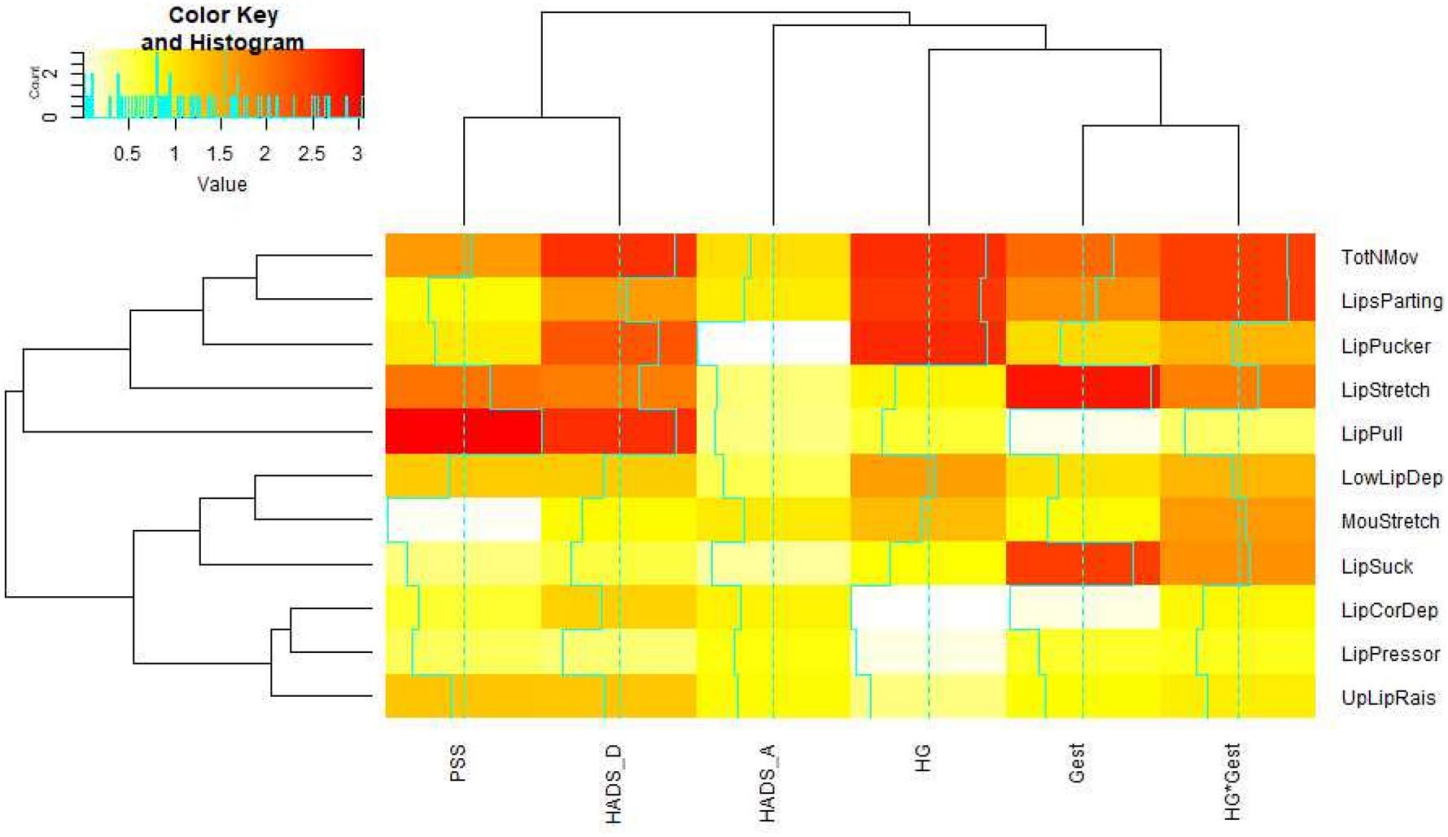
Heatmap with dendrogram for individual movements. The colour strength from red to white corresponds to the strength of effects with deep red the strongest effect, and white smallest effect. The strength of the effect can also be assessed through the light-blue step-shaped curves, with the vertical dashed lines indicating a *z* value of 2 on a standardized Gaussian scale. On the horizontal axis, Gest corresponds to gestational age, and HG × Gest to the interaction effect of gestational age and health status. On the vertical axis, the following abbreviations are used: *LowLipDep* lower lip depressor, *MouStretch* mouth stretch, *LipCorDep* lip corner depressor, *UpLipRais* upper lip raiser, *TotNMov* total number of movements

So far, we have discussed the total number of movements as the only response variable. However, we have specific measurements available on ten distinct movements, which are lips part, lip pucker, lip pull, upper lip raiser, lower lip depressor, lip corner depressor, lip pressor, mouth stretch, lip stretch, and lip suck. Based on the current small sample sizes, we would consider it as unscientific to report specific p values for each these (with multiple testings’ problems unavoidably arising), therefore we just present the results of this analysis in graphical form (Fig. 3) through a heatmap and dendrograms. Each row of the rows in the heatmap corresponds to a fitted model, with the respective movement type as response, and the variables PSS, HADS-A, HADS-D, HG, gestational age, as well the interaction term between the latter, as predictor terms. The colour in the heatmap represents the absolute *z* value of the estimated coefficients, where red means more evidence of relevance of each predictor variable for the respective movement type, and white less evidence. The graph also includes, in the first row of the heatmap, the total number of movements for reference.

Notably, the strongest HG effects are evident with the total number of movements, lip parting and lip puckering. Further promising directions for future research emerge from the graph; for instance, fetal lip pulling appears to be strongly related to maternal stress, anxiety, and depression. Lip pressing appears unexplained by any variable. The frequency of lip stretch, and lip suck is most strongly affected by the gestational week. It is useful to consider these results in conjunction with the movement-specific interaction plots for the effects of HG and gestational week (supplementary information S5). These plots show that, throughout all movement types, the most dominant feature is again the decrease in the number of movements from week 32 to week 36 for the HG group, corresponding to the generally darker shades in the last column of Fig. 3. This decrease is visible in S5 for seven out of ten movements in absolute terms, and for all ten movements relative to the healthy group.

## Discussion and conclusion

### HG affects fetal movement

In this study, we examined the effects of HG on the nutritional profile of the mother by testing maternal hair for carbon isotope ratios (δ^13^C) and nitrogen isotope ratios (δ^15^N) in hair reflecting maternal diet. At 32-weeks' gestation, fetuses with mothers having HG showed significantly higher rate of fetal movements compared with fetuses of healthy mothers. On the one hand, significantly decreased fetal movements signpost an increased risk of fetal growth restriction, stillbirth, preterm birth and the need of emergency caesarean section [36]. Donker et al. [37] suggested that a significant reduction in the range of different types of specific movement patterns, such as frequency of jaw opening or neck turn, are an indication that fine-grained movement profiles might be helpful in identifying distress. On the other hand, recent research by Heazell et al. [38] argues that maternal heightened anxiety, fetal seizures or unexplained fetal insult might lead to abnormally increased fetal movement profiles and stillbirth occurring after 37-weeks’ gestation. In the current study, movement profiles of fetuses of mothers with HG showed an increased movement profile at 32-weeks’ gestation and this was followed by the rapid decline of fine-grained mouth movements over the next 4 weeks to slightly (albeit nonsignificantly) below that of healthy fetuses. This movement profile at 36 weeks can be interpreted as either leading to normalized movement profiles or potentially to abnormally low frequencies of movements, which are themselves an indicator of an unhealthy development [36]. These interpretations might be distinguished using a scan later in pregnancy.

In sum, fine-grained movement analysis might be a helpful tool in identifying distress as well as normal development. Reissland et al. [15] and Castiello and Parma [39] argue that fetal movement profiles are potential tools to investigate prenatal cognition and the pre- to post-natal continuity of cognitive development including “anticipation” in the fetus. Hence, not only gross body movements, but also more fine-grained movement analyses might be usefully employed to assess healthy fetal development. In previous research, fetal movements tested included general body movements, startle and twitch movements, isolated limb movements, breathing movements, hiccups, isolated head and neck movements, sucking and swallowing, jaw movements (including yawning), hand–face contact, stretch and rotation [40, 41], as well as fine-grained fetal facial movements [42]. The analysis of gross body movements is a less conclusive measure of fetal distress compared to the fine-grained analysis utilised in this study. The heatmap (Fig. 3) indicates that there might be potential to use fine-grained movement analysis to highlight specific movements, such as lip parting and lip puckering, which may be predictive of fetal state. Furthermore, other specific movements (e.g., lip pull) might be influenced by maternal mental health which has previously been shown indicative of neurobehavioral development, such as increased eye-blink rate in fetuses of anxious mothers [5]. These specific movements could potentially be used as prenatal markers for adverse neurodevelopment.

### Hyperemesis is detectable in hair isotopes

Results indicated that the majority of mothers suffering from hyperemesis had either suspected or confirmed catabolic profiles in their first trimester hair samples. Our results confirm one other study showing that HG is evidenced in the stable isotopes of hair [8] by an increase in δ^15^N. HG mothers in the current study had experienced significant weight loss, which added to the power of our study, compared to the ‘restricted weight gain and/or weight loss’ previously studied [8], but our study lacks information from monitoring of diet and weight throughout pregnancy which could improve our understanding of isotopic changes. The current results suggest that with more extreme weight loss decreases in δ^13^C are also detectable. We also made no allowance for the fact that only 85–90% of scalp hairs are actively growing at any time and there is therefore smoothing of the isotopic signal when multiple hairs are sampled [43], but this could only be assessed using plucked rather than cut hairs. Catabolic and non-catabolic isotope profiles are currently identified by eye and classified in a binary fashion. However, the measurements are continuous data and, in the future, it would be useful to develop a quantified scale computed from the data to express graduations in dietary stress that mothers have experienced. This would allow more subtle effects on fetal development to be investigated, and the retrospective nature of their identification would reduce the confounding effect of unrecorded HG or morning sickness in studies of fetal development.

### HG and postnatal development

This interpretation of our prenatal data is strengthened by work on long-term effects of maternal HG on their offspring. Parker et al. [44] report long-term effects of children from 5–12 years in terms of poorer scores on several neurodevelopmental measures as well as psychosocial problems including internalizing and externalizing behaviours of children exposed to HG prenatally. The effects of HG seem to be long term beyond childhood. Mullin et al. [12] found when comparing emotional and behavioural outcomes of adults who had been exposed to HG during their fetal life compared with healthy pregnancies, those who were exposed to HG prenatally were significantly more likely to be diagnosed with depression, and anxiety as well as bipolar disorders in adulthood. Additionally, HG seemed to be a significant factor indicating that this condition needs to be treated not only prenatally, but that follow-up of the children might be called for. In sum, although the effects of HG are not apparent in terms of birthweight, Apgar scores or gestational age at delivery [45] there is evidence of postnatal effects, which warrant more investigation.

The current pilot study indicates that HG affects the normal development of fetuses, and that this is evident from as early as 32-weeks' gestation. More research is needed on the prevailing effects of this altered developmental trajectory. Additionally, a larger fetal study with a postnatal follow-up is needed to use these differentiated movement profiles in HG fetuses as potential markers for later neurodevelopmental outcomes.

## Electronic supplementary material

Below is the link to the electronic supplementary material.

Supplementary file1 (DOCX 230 kb)

## References

[1] Vohr BR, Poggi Davis E, Wanke CA, Krebs NF (2017). Neurodevelopment: the impact of nutrition and inflammation during preconception and pregnancy in low-resource settings. Pediatrics.

[2] Glover V, O’Donnell KJ, O’Connor TG, Fisher J (2018). Prenatal maternal stress, fetal programming, and mechanisms underlying later psychopathology- A global perspective. Dev Psychopathol.

[3] Barker D, Clark P (1997). Fetal undernutrition and disease in later life. Rev Reprod.

[4] Glover V (2015). Prenatal stress and its effects on the fetus and the child: possible underlying biological mechanisms. Adv Neurobiol.

[5] Reissland N, Froggatt S, Reames E, Girkin J (2018). Effects of maternal anxiety and depression on fetal neuro-development. J Affect Disord.

[6] Adair LS (2014). Longterm consequences of nutrition and growth in early childhood and possible preventive interventions. Nestle Nutr Inst Workshops Ser.

[7] Fall C, Yajnik CS, Rao S, Davies AA, Brown N, Farrant HJ (2003). Micronutrients and fetal growth. J Nutr.

[8] Fuller BT, Fuller JL, Sage NE (2005). Nitrogen balance and δ^15^N: why you're not what you eat during nutritional stress. Rapid Commun Mass Spectrom.

[9] Fejzo MS, Schoenberg FP, Macgibbon K (2016). Long-term health effects in children exposed in utero to hyperemesis gravidarum. Clin Obstet Gynecol Reprod Med.

[10] Fejzo M, Kam A, Laguna A, MacGibbon K, Mullin P (2019). Analysis of neurodevelopmental delay in children exposed in utero to hyperemesis gravidarum reveals increased reporting of autism spectrum disorder. Reprod Toxicol.

[11] Fejzo MS, Magtira A, Schoenberg FP (2015). Neurodevelopmental delay in children exposed in utero to hyperemesis gravidarum. Eur J Obstet Gynecol Reprod Biol.

[12] Mullin PM, Bray A, Schoenberg F, MacGibbon KW (2011). Prenatal exposure to hyperemesis gravidarum linked to increased risk of psychological and behavioral disorders in adulthood. J Dev Orig Health Dis.

[13] Lacasse A, Rey E, Ferreira E (2009). Epidemiology of nausea and vomiting of pregnancy: prevalence, severity, determinants, and the importance of race/ethnicity. BMC Pregnancy Childbirth.

[14] Zhang J, Cai WW (1991). Severe vomiting during pregnancy: antenatal correlates and fetal outcomes. Epidemiology.

[15] Reissland N (2014). What the fetal face can tell us a discussion of the evidence, implications and potential for further research. Donald School J Ultrasound Obstet Gynecol.

[16] Reissland N, Francis B, Buttanshaw L, Reissland N, Kisilevsky B (2016). The fetal observable movement system (FOMS). Fetal development research on brain and behavior, environmental influences, and emerging technologies.

[17] Kurjak A, Carrera JM, Stanojevic M (2004). The role of 4D ultrasound in the neurological assessment of early human development. Ultrasound Rev Obstet Gynecol.

[18] AboEllail MAM, Hata T (2017). Fetal face as important indicator of fetal brain function. J Perinat Med.

[19] Reissland N, Francis B, Buttanshaw L (2016). Do fetuses move their lips to the sound that they hear?. Pilot Feasibility Stud.

[20] Coplen TB (2011). Guidelines and recommended terms for expression of stable-isotope-ratio and gas-ratio measurement results. Rapid Commun Mass Spectrom.

[21] Lee-Thorp JA, Sealy JC, van der Merwe NJ (1989). Stable carbon isotope ratio differences between bone collagen and bone apatite and their relationship to diet. J Archaeol Sci.

[22] Mekota A-M, Grupe G, Ufer S, Cuntz U (2006). Serial analysis of stable nitrogen and carbon isotopes in hair: monitoring starvation and recovery phases of patients suffering from anorexia nervosa. Rapid Commun Mass Spectrom.

[23] Mekota AM, Grupe G, Ufer S, Cuntz U (2009). Identifying starvation episodes using stable isotopes in hair. Rechtsmedizin.

[24] Neuberger FM, Jopp E, Graw M, Püschel K, Grupe G (2013). Signs of malnutrition and starvation—reconstruction of nutritional life histories by serial isotopic analyses of hair. Forensic Sci Int.

[25] Cohen S, Kamarck T, Mermelstein R (1983). A global measure of perceived stress. J Health Soc Behav.

[26] Cohen S, Williamson G, Spacapan S, Oskamp S (1988). Perceived Stress in a Probability Sample of the United States. The Social Psychology of Health.

[27] Zigmond AS, Snaith RP (1983). The hospital anxiety and depression scale. Acta Psychiatrica Scandinavia.

[28] Koren G, Boskovic R, Hard M (2002). (PUQE) (pregnancy-unique quantification of emesis and nausea) scoring system for nausea and vomiting of pregnancy. Am J Obstet Gynecol.

[29] Condon JT, Cokindale CJ (1998). The assessment of parent-to-infant attachment: development of a self-report questionnaire instrument. J Reprod Infant Psychol.

[30] O'Connell TC, Hedges REM (1999). Investigations into the effect of diet on modern human hair isotopic values. Am J Phys Anthropol.

[31] O'Connell TC, Hedges REM, Healey MA, Simpson AHRW (2001). Isotopic comparison of hair, nail and bone: modern analyses. J Archaeol Sci.

[32] Reissland N, Francis BJ, Mason J (2012). Development of fetal yawn compared with non-yawn mouth openings from 24–36 weeks gestation. PLoS ONE.

[33] Reissland N, Francis B, Aydin E (2013). The development of anticipation in the fetus: a longitudinal account of human fetal mouth movements in reaction to and anticipation of touch. Dev Psychobiol.

[34] Brooks ME, Kristensen K, van Benthem KJ, et al (2017) Modelling zero-inflated count data with glmmTMB. bioRxiv, https://www.biorxiv.org/content/10.1101/132753v1. Accessed 26 Feb 2020

[35] Calhoun P (2016) Exact: unconditional exact test. R package version 1.7. Cran.R. https://CRAN.R-project.org/package=Exact29. Accessed June 2019

[36] Olesen AG, Svare JA (2007). Decreased fetal movements: background, assessment, and clinical management. Acta Obstetrica Gynecologica Scandinavia.

[37] Donker ME, Eijckelhof BH, Tan G, de Vries JI (2009). Serial postural and motor assessment of fetal akinesia deformation sequence (FADS). Early Human Dev.

[38] Heazell AEP, Stacey T, O’Brien LM, Mitchell EA, Warland J (2018). Excessive fetal movements are a sign of fetal compromise which merits further examination. Med Hypotheses.

[39] Castiello U, Parma V (2018). Fetal kinematics: basic outcomes and translational outlook. ACS Chem Neurosci.

[40] Kurjak A, Stanojevic M, Andonotopo W (2005). Fetal behavior assessed in all three trimesters of normal pregnancy by four-dimensional ultrasonography. Croat Med J.

[41] De Vries JIP, Visser GHA, Prechtl HFR (1982). The emergence of fetal behaviour. I Qualitative aspects Early Human Development.

[42] Reissland N, Francis B, Mason J, Lincoln K (2011). Do facial expressions develop before birth?. PLoS ONE.

[43] Williams LJ, White CD, Longstaffe FJ (2011). Improving stable isotopic interpretations made from human hair through reduction of growth cycle error. Am J Phys Anthropol.

[44] Parker SE, Starr JR, Collett BR (2014). Nausea and vomiting during pregnancy and neurodevelopmental outcomes in offspring. Paediatr Perinat Epidemiol.

[45] Agmon N, Sade S, Periente G (2019). Hyperemesis gravidarum and adverse pregnancy outcomes. Arch Gynecol Obstet.

