# Supplementary material for: Prenatal effects of maternal nutritional stress and mental health on the fetal movement profile
Source: Arch Gynecol Obstet. 2020 May 14;302(1):65–75. doi: 10.1007/s00404-020-05571-w (PMC7266842; doi:10.1007/s00404-020-05571-w)

**Prenatal effects of maternal nutritional stress and mental health on the fetal movement profile**

**Supplementary information**

**Supplementary information S1: Isotope measurement results**

| **Sample Code** | **Nitrogen Content (%)** | **δ^15^N_AIR_ (‰)** | **Carbon Content (%)** | **δ^13^C_V-PDB_ (‰)** | **C:N ratio (atomic)** |
| --- | --- | --- | --- | --- | --- |
| 3920-01 | 16.38 | 8.88 | 47.12 | -21.49 | 2.46 |
| 3920-02 | 15.96 | 8.94 | 46.20 | -21.38 | 2.48 |
| 3920-03 | 13.81 | 9.11 | 40.98 | -21.48 | 2.54 |
| 3920-04 | 14.75 | 8.99 | 44.67 | -21.54 | 2.60 |
| 3920-05 | 14.61 | 8.73 | 44.82 | -21.81 | 2.63 |
| " | 14.43 | 8.74 | 44.72 | -21.80 | 2.66 |
| 3920-06 | 15.74 | 8.75 | 48.14 | -21.73 | 2.62 |
| 3920-07 | 16.10 | 8.88 | 48.51 | -21.72 | 2.58 |
| 3920-08 | 15.12 | 8.90 | 45.48 | -21.90 | 2.58 |
| 3920-09 | 15.19 | 8.89 | 45.63 | -21.86 | 2.57 |
| 3920-10 | 15.97 | 8.88 | 47.99 | -21.84 | 2.58 |
| 3921-01 | 15.56 | 8.80 | 43.95 | -21.68 | 2.42 |
| 3921-02 | 16.03 | 8.89 | 45.70 | -21.79 | 2.44 |
| 3921-03 | 16.60 | 8.88 | 47.15 | -21.58 | 2.43 |
| 3921-04 | 13.40 | 9.05 | 38.48 | -21.83 | 2.46 |
| 3921-05 | 13.82 | 8.96 | 40.03 | -21.59 | 2.48 |
| " | 15.60 | 8.95 | 46.30 | -21.56 | 2.54 |
| 3921-06 | 15.52 | 9.08 | 44.52 | -21.43 | 2.46 |
| 3921-07 | 15.66 | 9.18 | 44.72 | -21.57 | 2.45 |
| 3921-08 | 17.11 | 9.15 | 49.33 | -21.47 | 2.47 |
| 3921-09 | 16.89 | 9.24 | 49.21 | -21.39 | 2.50 |
| 3921-10 | 14.85 | 9.03 | 42.93 | -21.81 | 2.48 |
| " | 15.44 | 9.08 | 44.90 | -21.88 | 2.49 |
| 3922-01 | 16.09 | 9.36 | 46.02 | -21.69 | 2.45 |
| 3922-02 | 15.98 | 9.39 | 46.05 | -21.56 | 2.47 |
| 3922-03 | 16.54 | 9.36 | 47.65 | -21.55 | 2.47 |
| 3922-04 | 15.46 | 9.47 | 51.43 | -21.85 | 2.85 |
| 3922-05 | 14.03 | 9.52 | 47.26 | -21.86 | 2.89 |
| " | 15.38 | 9.55 | 46.97 | -21.67 | 2.62 |
| 3922-06 | 15.86 | 9.62 | 46.45 | -21.72 | 2.51 |
| 3922-07 | 14.49 | 9.73 | 42.10 | -21.72 | 2.49 |
| 3922-08 | 16.05 | 9.76 | 46.39 | -21.61 | 2.48 |
| 3922-09 | 16.49 | 9.82 | 48.03 | -21.78 | 2.50 |
| 3922-10 | 16.54 | 9.82 | 48.29 | -21.92 | 2.50 |
| " | 14.92 | 9.88 | 43.49 | -21.91 | 2.50 |
| 3923-01 | 14.66 | 8.67 | 42.23 | -21.72 | 2.47 |
| 3923-02 | 15.16 | 8.74 | 43.87 | -21.62 | 2.48 |
| 3923-03 | 15.98 | 8.86 | 46.45 | -21.64 | 2.49 |
| 3923-04 | 16.05 | 8.92 | 47.36 | -21.52 | 2.53 |
| 3923-05 | 15.10 | 8.98 | 48.34 | -21.67 | 2.74 |
| " | 15.15 | 9.16 | 51.25 | -21.83 | 2.90 |
| 3923-06 | 16.38 | 9.20 | 46.84 | -21.52 | 2.45 |
| 3923-07 | 15.07 | 9.14 | 43.30 | -21.65 | 2.46 |
| 3923-08 | 18.72 | 9.14 | 53.87 | -21.74 | 2.47 |
| 3923-09 | 16.07 | 9.15 | 46.19 | -21.81 | 2.46 |
| 3923-10 | 17.07 | 9.12 | 49.07 | -21.80 | 2.46 |
| 3924-01 | 17.58 | 8.47 | 49.88 | -21.41 | 2.43 |
| " | 16.30 | 8.49 | 46.61 | -21.37 | 2.45 |
| 3924-02 | 16.65 | 8.33 | 47.55 | -21.11 | 2.45 |
| 3924-03 | 16.27 | 8.58 | 46.64 | -21.31 | 2.46 |
| 3924-04 | 16.03 | 8.89 | 45.55 | -21.15 | 2.44 |
| 3924-05 | 15.93 | 9.21 | 45.42 | -21.15 | 2.44 |
| " | 15.81 | 9.16 | 45.02 | -21.11 | 2.44 |
| 3924-06 | 16.14 | 9.30 | 46.28 | -21.17 | 2.46 |
| 3924-07 | 15.19 | 9.27 | 43.94 | -21.00 | 2.48 |
| 3924-08 | 13.40 | 9.14 | 38.90 | -20.96 | 2.49 |
| 3924-09 | 16.03 | 9.22 | 46.35 | -21.57 | 2.48 |
| 3924-10 | 13.70 | 9.31 | 39.97 | -21.60 | 2.50 |
| " | 15.59 | 9.28 | 45.45 | -21.69 | 2.50 |
| 3925-01 | 15.41 | 7.81 | 44.26 | -21.44 | 2.46 |
| 3925-02 | 15.31 | 8.19 | 44.02 | -21.33 | 2.47 |
| 3925-03 | 15.81 | 8.08 | 45.95 | -21.20 | 2.49 |
| 3925-04 | 15.48 | 8.27 | 45.00 | -21.36 | 2.49 |
| 3925-05 | 15.01 | 8.37 | 43.60 | -21.27 | 2.49 |
| " | 15.31 | 8.33 | 44.65 | -21.16 | 2.50 |
| 3925-06 | 15.77 | 8.39 | 45.42 | -21.26 | 2.47 |
| 3925-07 | 15.58 | 8.44 | 44.93 | -21.20 | 2.47 |
| 3925-08 | 18.01 | 8.21 | 52.44 | -21.35 | 2.50 |
| 3925-09 | 15.13 | 7.97 | 44.66 | -21.57 | 2.53 |
| 3925-10 | 15.81 | 7.75 | 46.97 | -21.64 | 2.55 |
| " | 14.84 | 7.86 | 44.38 | -21.59 | 2.56 |
| 3926-01 | 18.22 | 9.22 | 52.71 | -21.34 | 2.48 |
| 3926-02 | 16.23 | 9.44 | 47.38 | -21.44 | 2.50 |
| 3926-03 | 15.94 | 9.52 | 46.89 | -21.50 | 2.52 |
| 3926-04 | 16.17 | 9.40 | 48.11 | -21.59 | 2.55 |
| 3926-05 | 15.66 | 9.37 | 45.91 | -21.65 | 2.51 |
| " | 13.95 | 9.33 | 40.86 | -21.50 | 2.51 |
| 3926-06 | 16.29 | 9.49 | 47.90 | -21.65 | 2.52 |
| 3926-07 | 16.86 | 9.71 | 48.78 | -21.71 | 2.48 |
| 3926-08 | 15.01 | 9.92 | 43.81 | -21.68 | 2.50 |
| 3926-09 | 15.87 | 9.53 | 46.36 | -21.75 | 2.50 |
| 3926-10 | 16.11 | 9.58 | 47.08 | -21.88 | 2.51 |
| " | 16.52 | 9.57 | 48.11 | -21.90 | 2.50 |
| 3927-01 | 14.60 | 8.53 | 41.79 | -21.53 | 2.45 |
| 3927-02 | 15.39 | 8.71 | 44.34 | -21.54 | 2.47 |
| 3927-03 | 16.12 | 8.74 | 46.54 | -21.62 | 2.47 |
| 3927-04 | 16.26 | 8.73 | 47.01 | -21.71 | 2.48 |
| 3927-05 | 15.61 | 8.84 | 45.34 | -21.62 | 2.49 |
| " | 15.40 | 8.83 | 44.63 | -21.53 | 2.48 |
| 3927-06 | 15.35 | 8.98 | 44.72 | -21.71 | 2.50 |
| 3927-07 | 15.84 | 9.53 | 45.74 | -21.51 | 2.48 |
| 3927-08 | 16.76 | 9.71 | 48.39 | -21.70 | 2.47 |
| 3927-09 | 15.40 | 9.59 | 44.63 | -21.76 | 2.48 |
| 3927-10 | 15.99 | 9.45 | 46.36 | -21.95 | 2.48 |
| " | 15.59 | 9.45 | 45.32 | -21.88 | 2.49 |
| 3928-01 | 16.12 | 8.79 | 45.94 | -21.06 | 2.44 |
| 3928-02 | 16.92 | 8.79 | 48.22 | -21.07 | 2.44 |
| 3928-03 | 15.52 | 8.93 | 44.38 | -20.99 | 2.45 |
| 3928-04 | 15.34 | 9.08 | 44.02 | -21.24 | 2.46 |
| 3928-05 | 14.48 | 9.17 | 41.58 | -21.20 | 2.46 |
| " | 15.43 | 9.15 | 44.26 | -21.29 | 2.46 |
| 3928-06 | 15.43 | 9.34 | 44.26 | -21.11 | 2.46 |
| 3928-07 | 15.67 | 9.48 | 45.14 | -21.09 | 2.47 |
| 3928-08 | 14.20 | 9.41 | 40.94 | -21.29 | 2.47 |
| 3928-09 | 15.34 | 9.30 | 44.22 | -21.34 | 2.47 |
| 3928-10 | 15.41 | 9.29 | 44.30 | -21.40 | 2.46 |
| " | 15.44 | 9.25 | 44.54 | -21.26 | 2.47 |
| 3929-01 | 16.27 | 8.59 | 46.48 | -21.26 | 2.45 |
| 3929-02 | 15.35 | 8.52 | 43.79 | -21.26 | 2.45 |
| 3929-04 | 15.11 | 8.50 | 44.85 | -21.64 | 2.54 |
| 3929-05 | 14.58 | 8.88 | 43.15 | -21.64 | 2.54 |
| " | 15.52 | 8.86 | 46.14 | -21.70 | 2.55 |
| 3929-06 | 13.91 | 9.45 | 41.84 | -21.71 | 2.58 |
| 3929-07 | 14.53 | 9.59 | 43.32 | -21.57 | 2.56 |
| 3929-08 | 14.11 | 9.51 | 41.65 | -21.76 | 2.53 |
| 3929-09 | 14.52 | 9.56 | 42.93 | -21.74 | 2.53 |
| 3929-10 | 16.68 | 9.56 | 49.42 | -21.88 | 2.54 |
| " | 14.18 | 9.58 | 42.27 | -21.89 | 2.56 |
| 3930-01 | 14.87 | 8.93 | 46.35 | -22.16 | 2.67 |
| 3930-02 | 13.95 | 8.98 | 42.06 | -21.78 | 2.58 |
| 3930-03 | 14.92 | 8.98 | 43.52 | -21.39 | 2.50 |
| 3930-04 | 15.52 | 9.17 | 44.85 | -21.11 | 2.48 |
| 3930-05 | 16.14 | 9.43 | 46.60 | -21.41 | 2.47 |
| " | 16.08 | 9.54 | 47.62 | -21.51 | 2.54 |
| 3930-06 | 16.30 | 9.51 | 48.53 | -21.83 | 2.55 |
| 3930-07 | 16.05 | 9.72 | 47.16 | -21.71 | 2.52 |
| 3930-08 | 15.94 | 9.50 | 47.37 | -21.89 | 2.55 |
| 3930-09 | 15.13 | 9.51 | 45.42 | -22.09 | 2.57 |
| 3930-10 | 14.79 | 9.38 | 44.78 | -22.08 | 2.59 |
| " | 14.11 | 9.35 | 43.74 | -22.20 | 2.66 |
| 3931-01 | 15.51 | 8.87 | 45.08 | -21.75 | 2.49 |
| 3931-02 | 15.58 | 9.04 | 45.47 | -21.72 | 2.50 |
| 3931-03 | 15.50 | 9.20 | 46.20 | -21.53 | 2.56 |
| 3931-04 | 15.77 | 9.37 | 46.12 | -21.81 | 2.51 |
| 3931-05 | 16.14 | 9.45 | 47.38 | -21.89 | 2.52 |
| " | 16.02 | 9.48 | 47.17 | -21.95 | 2.52 |
| 3931-06 | 15.67 | 9.39 | 47.08 | -21.91 | 2.57 |
| 3931-07 | 16.03 | 9.46 | 47.80 | -21.93 | 2.56 |
| 3931-08 | 17.09 | 9.43 | 50.47 | -21.93 | 2.53 |
| 3931-09 | 17.34 | 9.45 | 51.68 | -22.02 | 2.55 |
| 3931-10 | 16.28 | 9.38 | 48.83 | -22.00 | 2.57 |
| " | 14.35 | 9.37 | 43.53 | -22.03 | 2.60 |
| 3932-01 | 16.68 | 9.08 | 50.17 | -21.35 | 2.58 |
| 3932-02 | 15.85 | 9.26 | 47.37 | -21.24 | 2.56 |
| 3932-03 | 15.15 | 9.33 | 46.07 | -21.42 | 2.61 |
| 3932-04 | 14.85 | 9.31 | 44.28 | -21.28 | 2.56 |
| 3932-05 | 15.72 | 9.28 | 47.47 | -21.40 | 2.59 |
| " | 14.50 | 9.34 | 44.51 | -21.39 | 2.63 |
| 3932-06 | 14.97 | 9.42 | 45.64 | -21.71 | 2.61 |
| 3932-07 | 16.28 | 9.37 | 48.83 | -21.72 | 2.57 |
| 3932-08 | 14.54 | 9.34 | 43.56 | -21.73 | 2.57 |
| 3932-09 | 15.71 | 9.42 | 46.74 | -21.65 | 2.55 |
| 3932-10 | 16.21 | 9.37 | 48.44 | -21.64 | 2.56 |
| 3933-01 | 15.46 | 8.81 | 45.39 | -21.33 | 2.52 |
| 3933-02 | 16.17 | 8.99 | 46.86 | -21.33 | 2.48 |
| 3933-03 | 16.06 | 8.88 | 47.67 | -21.61 | 2.54 |
| 3933-04 | 16.01 | 9.09 | 46.92 | -21.35 | 2.51 |
| 3933-05 | 16.29 | 9.27 | 48.10 | -21.38 | 2.53 |
| " | 15.67 | 9.23 | 46.31 | -21.04 | 2.53 |
| 3933-06 | 12.63 | 9.52 | 36.92 | -21.21 | 2.51 |
| 3933-07 | 15.93 | 9.60 | 47.22 | -21.31 | 2.54 |
| 3933-08 | 16.03 | 9.56 | 47.47 | -21.31 | 2.54 |
| 3933-09 | 15.34 | 9.53 | 45.56 | -21.52 | 2.55 |
| 3933-10 | 16.73 | 9.52 | 50.26 | -21.85 | 2.58 |
| " | 16.21 | 9.59 | 48.84 | -21.68 | 2.58 |
| 3934-01 | 14.96 | 7.87 | 43.16 | -22.12 | 2.47 |
| 3934-02 | 14.10 | 8.07 | 40.83 | -22.17 | 2.48 |
| 3934-03 | 15.79 | 8.35 | 45.43 | -21.97 | 2.47 |
| 3934-04 | 15.46 | 8.70 | 44.20 | -21.77 | 2.45 |
| 3934-05 | 15.15 | 8.98 | 43.49 | -21.90 | 2.46 |
| " | 15.08 | 8.90 | 43.21 | -21.94 | 2.46 |
| 3934-06 | 14.52 | 9.08 | 42.10 | -21.97 | 2.49 |
| 3934-07 | 15.66 | 8.90 | 45.37 | -21.91 | 2.48 |
| 3934-08 | 16.41 | 8.81 | 47.10 | -21.93 | 2.46 |
| 3934-09 | 15.93 | 8.78 | 45.94 | -21.96 | 2.47 |
| 3934-10 | 15.98 | 8.80 | 46.16 | -22.10 | 2.48 |
| " | 15.64 | 8.79 | 45.31 | -22.02 | 2.48 |
| 3935-01 | 17.70 | 8.97 | 51.21 | -21.31 | 2.48 |
| 3935-02 | 16.62 | 9.06 | 47.89 | -21.34 | 2.47 |
| 3935-03 | 15.75 | 9.11 | 45.71 | -21.35 | 2.49 |
| 3935-04 | 14.95 | 9.24 | 43.28 | -21.32 | 2.48 |
| 3935-05 | 15.16 | 9.52 | 44.19 | -21.51 | 2.50 |
| " | 14.54 | 9.50 | 42.33 | -21.54 | 2.50 |
| 3935-06 | 15.87 | 9.68 | 46.10 | -21.56 | 2.49 |
| 3935-07 | 14.55 | 9.56 | 56.12 | -23.12 | 3.31 |
| 3935-08 | 14.94 | 9.45 | 43.45 | -21.72 | 2.49 |
| 3935-09 | 15.17 | 9.45 | 44.29 | -21.74 | 2.50 |
| 3935-10 | 15.46 | 9.47 | 44.83 | -21.77 | 2.49 |
| " | 15.37 | 9.39 | 45.06 | -21.73 | 2.51 |
| 3936-01 | 15.85 | 8.49 | 47.22 | -21.27 | 2.55 |
| 3936-02 | 14.44 | 8.82 | 42.08 | -21.35 | 2.50 |
| 3936-03 | 16.03 | 8.86 | 46.10 | -21.27 | 2.47 |
| 3936-04 | 16.21 | 8.84 | 46.57 | -21.32 | 2.46 |
| 3936-05 | 16.05 | 8.92 | 46.20 | -21.29 | 2.47 |
| " | 16.30 | 9.02 | 46.96 | -21.25 | 2.47 |
| 3936-06 | 14.65 | 9.13 | 42.53 | -21.36 | 2.49 |
| 3936-07 | 17.01 | 9.30 | 48.89 | -21.51 | 2.46 |
| 3936-08 | 15.80 | 9.32 | 45.62 | -21.44 | 2.47 |
| 3936-09 | 14.93 | 9.35 | 43.32 | -21.43 | 2.49 |
| 3936-10 | 16.96 | 9.38 | 49.84 | -21.52 | 2.52 |
| " | 16.59 | 9.37 | 48.18 | -21.36 | 2.49 |
| 3937-01 | 15.45 | 9.75 | 44.86 | -20.99 | 2.49 |
| 3937-02 | 15.42 | 9.66 | 44.89 | -21.14 | 2.50 |
| 3937-03 | 14.63 | 9.73 | 42.84 | -21.23 | 2.51 |
| 3937-04 | 13.79 | 9.93 | 41.23 | -21.47 | 2.56 |
| 3937-05 | 15.62 | 9.95 | 45.82 | -21.53 | 2.51 |
| " | 14.46 | 9.83 | 42.69 | -21.51 | 2.53 |
| 3937-06 | 14.80 | 9.93 | 43.05 | -21.47 | 2.49 |
| 3937-07 | 14.00 | 9.93 | 41.00 | -21.64 | 2.51 |
| 3937-08 | 14.57 | 9.94 | 43.03 | -21.54 | 2.53 |
| 3937-09 | 15.19 | 9.69 | 44.48 | -21.38 | 2.51 |
| 3937-10 | 15.09 | 9.67 | 44.11 | -21.60 | 2.51 |
| " | 14.15 | 9.65 | 41.55 | -21.56 | 2.52 |
| 3938-01 | 15.63 | 8.50 | 44.61 | -21.00 | 2.45 |
| 3938-02 | 15.62 | 8.58 | 44.49 | -21.15 | 2.44 |
| 3938-03 | 15.72 | 8.41 | 44.96 | -21.11 | 2.45 |
| 3938-04 | 14.90 | 8.42 | 43.14 | -21.07 | 2.48 |
| 3938-05 | 14.06 | 8.75 | 41.61 | -20.91 | 2.54 |
| " | 15.00 | 8.68 | 43.86 | -20.90 | 2.51 |
| 3938-06 | 15.26 | 9.28 | 44.95 | -20.77 | 2.52 |
| 3938-07 | 15.81 | 9.35 | 47.98 | -21.04 | 2.60 |
| 3938-08 | 14.07 | 9.23 | 43.60 | -21.22 | 2.66 |
| 3938-09 | 15.20 | 8.93 | 46.65 | -21.35 | 2.63 |
| 3938-10 | 15.19 | 8.74 | 46.33 | -21.34 | 2.61 |
| " | 15.35 | 8.86 | 46.89 | -21.38 | 2.62 |
| 3939-01 | 16.11 | 8.95 | 46.33 | -21.01 | 2.47 |
| 3939-02 | 14.32 | 8.81 | 42.25 | -21.43 | 2.53 |
| 3939-03 | 15.08 | 9.04 | 44.08 | -21.50 | 2.51 |
| 3939-04 | 15.13 | 9.09 | 44.19 | -21.43 | 2.50 |
| 3939-05 | 15.08 | 9.26 | 44.35 | -21.31 | 2.52 |
| " | 15.78 | 9.42 | 46.05 | -21.34 | 2.50 |
| 3939-06 | 16.31 | 9.42 | 47.32 | -21.34 | 2.49 |
| 3939-07 | 16.07 | 9.48 | 46.47 | -21.52 | 2.48 |
| 3939-08 | 15.03 | 9.36 | 44.03 | -21.41 | 2.51 |
| 3939-09 | 14.97 | 9.28 | 44.19 | -21.66 | 2.53 |
| 3939-10 | 14.97 | 9.36 | 43.92 | -21.46 | 2.51 |
| " | 14.74 | 9.38 | 43.17 | -21.58 | 2.51 |
| 3939A-01 | 16.47 | 8.89 | 47.43 | -21.53 | 2.47 |
| 3939A-02 | 15.97 | 9.11 | 46.42 | -21.57 | 2.49 |
| 3939A-03 | 15.62 | 9.06 | 45.73 | -21.42 | 2.51 |
| 3939A-04 | 15.26 | 9.10 | 44.56 | -21.23 | 2.50 |
| 3939A-05 | 16.27 | 9.10 | 47.64 | -21.31 | 2.51 |
| " | 16.83 | 9.21 | 48.93 | -21.44 | 2.49 |
| 3939A-06 | 15.68 | 9.38 | 45.14 | -21.35 | 2.47 |
| 3939A-07 | 16.11 | 9.68 | 46.80 | -21.55 | 2.49 |
| 3939A-08 | 15.97 | 9.89 | 46.78 | -21.62 | 2.51 |
| 3939A-09 | 14.91 | 9.84 | 43.98 | -21.65 | 2.53 |
| 3939A-10 | 16.08 | 9.71 | 46.62 | -21.52 | 2.49 |
| " | 15.86 | 9.58 | 46.35 | -21.47 | 2.50 |

**Supplementary information S2: Isotopic profiles for all twelve women**


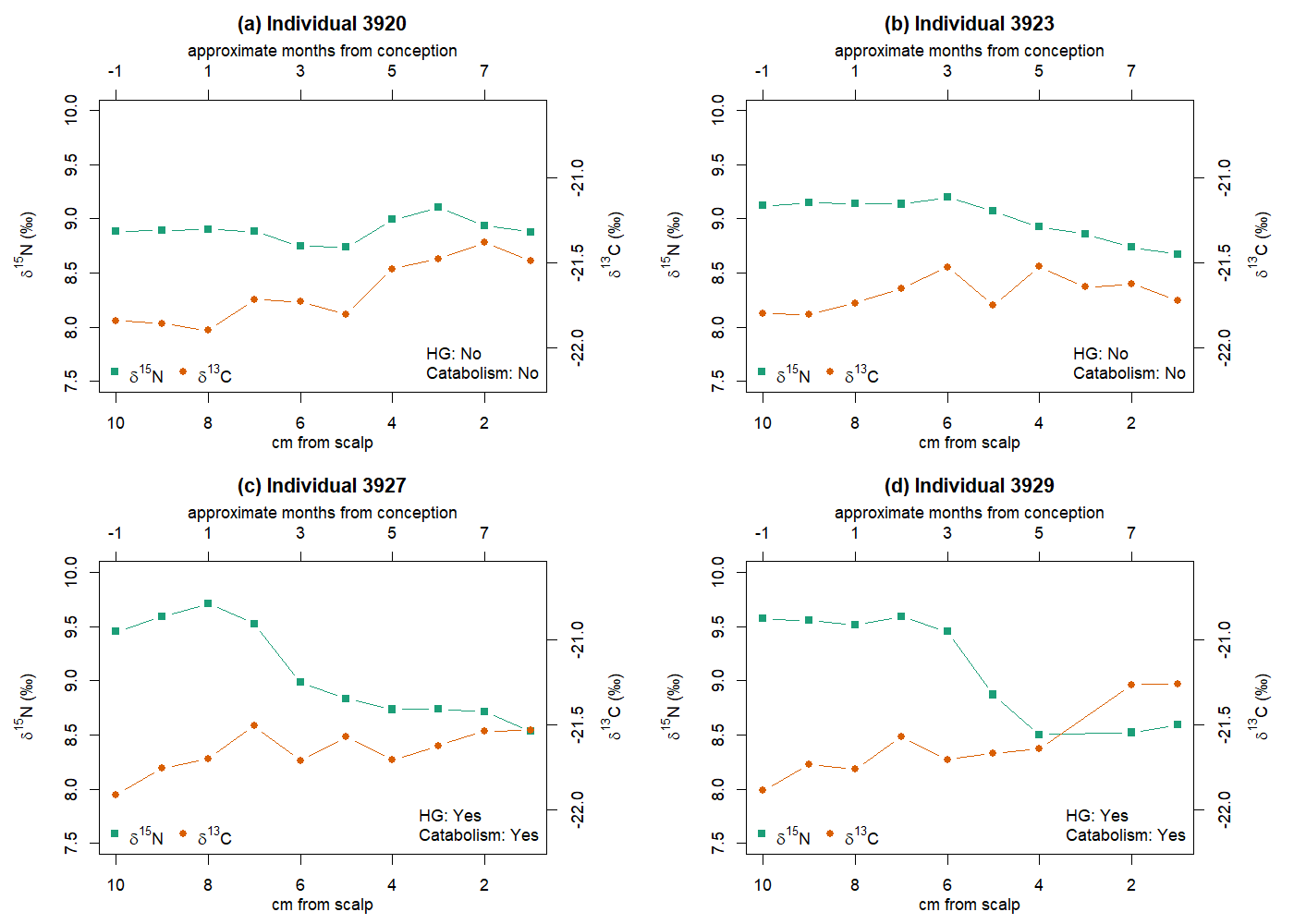


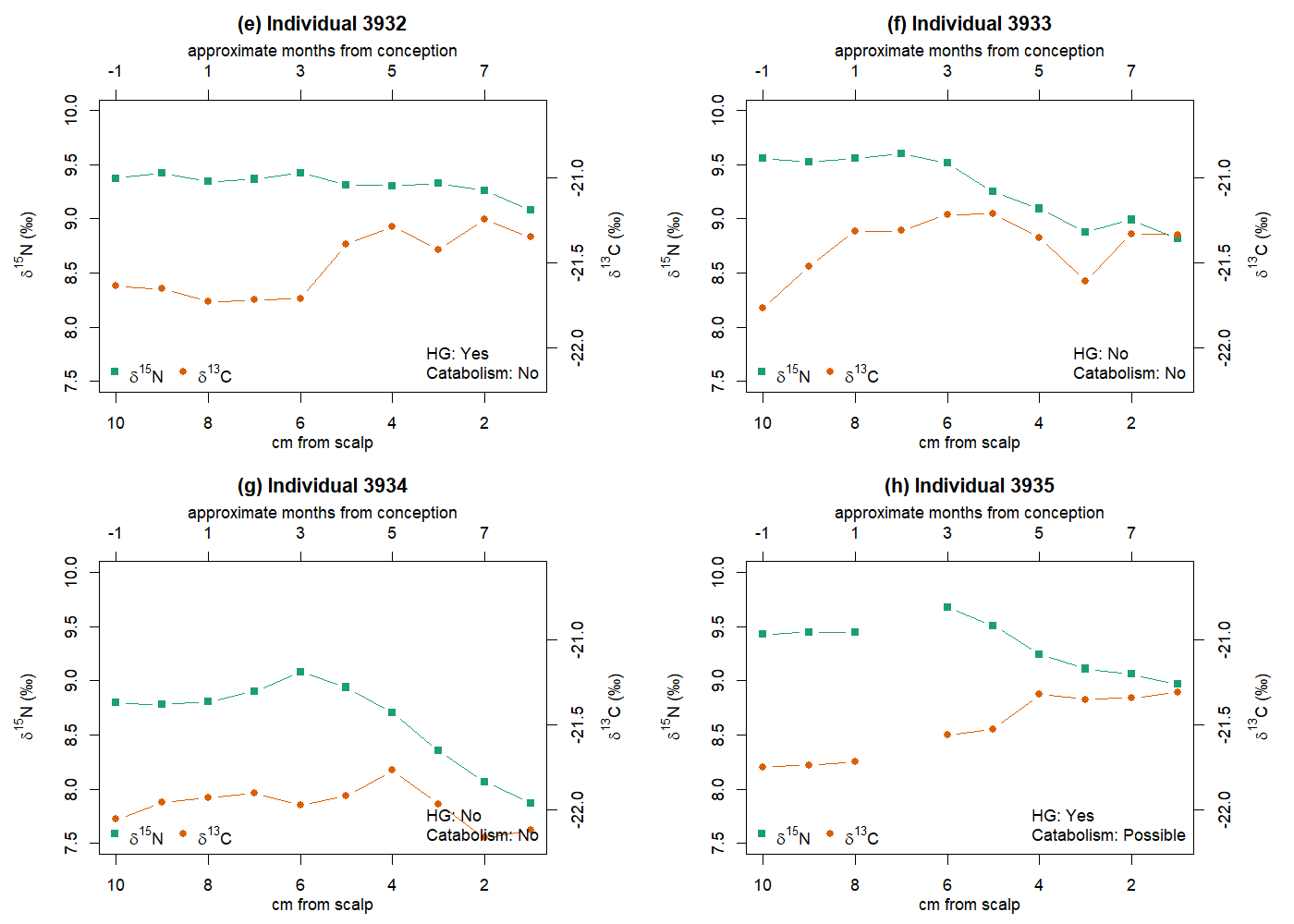


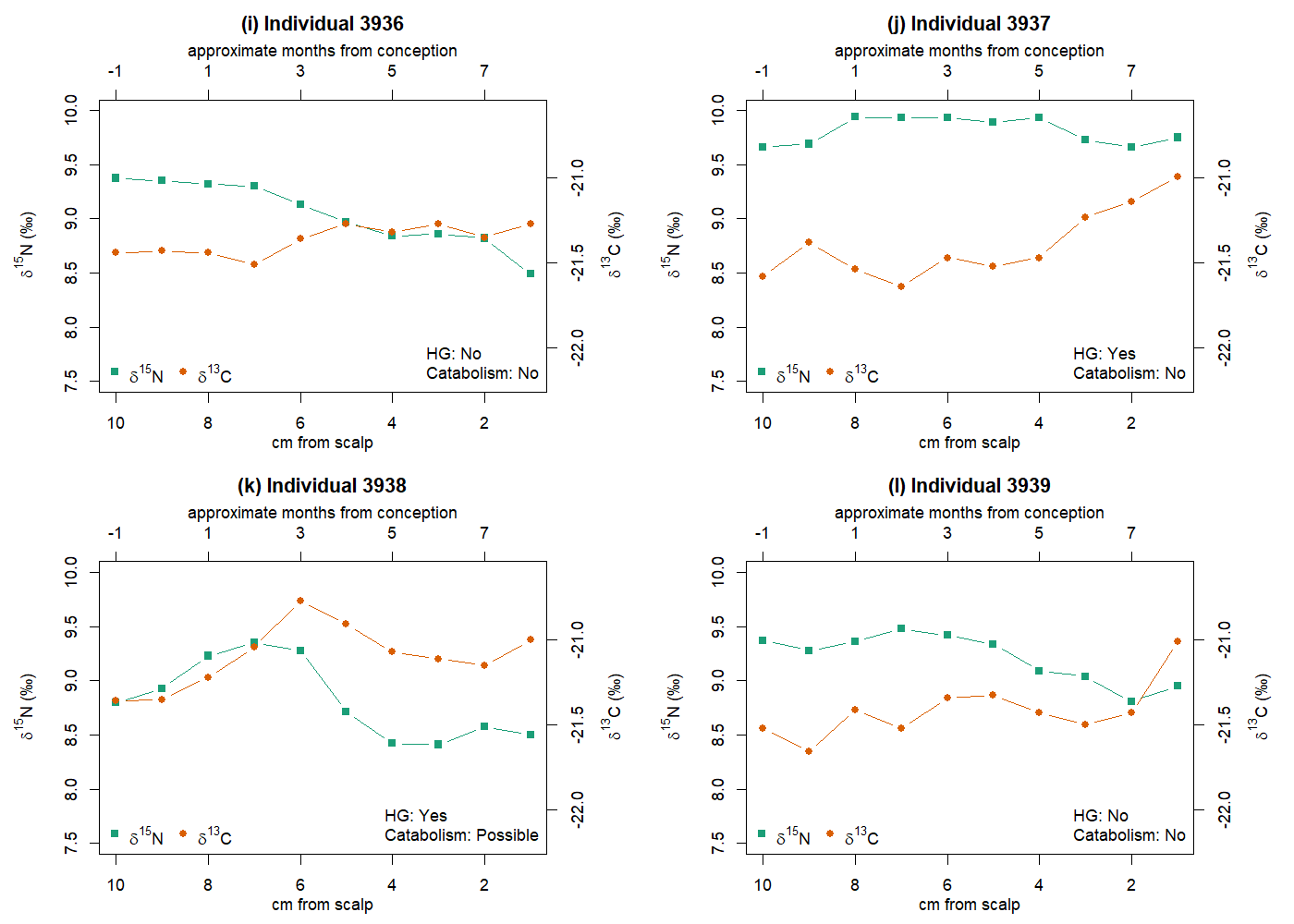


**Supplementary information S3: Excluded variables**

As indicated in Tables 1 and 2 in the main paper, several variables had been collected in this study which were not included into models (H) or (C). We give here some justification for their non-inclusion.

Note firstly that these variables cannot be added to (H) or (C) jointly since the data do not possess sufficiently many degrees of freedom. One can, however, include one variable at a time. The table below reports the p-values when adding each specific variable individually to model (H). The left hand part considers all variables except education, ordered by decreasing significance. The right hand part considers the three indicator variables required to fit model the 4-level factor for education (where, GCSE takes the role of the reference category, and all three indicators are included jointly into the model).

| Variable | p-value | Variable | p-value |
| --- | --- | --- | --- |
| ApgarScore5min | 0.0480 | LevelofEducation |  |
| MaternalAge | 0.1654 | College/A-level | 0.5152 |
| HeadCircumference | 0.2134 | Degree | 0.0833 |
| Birthweight | 0.2414 | Masters/Postgrad | 0.9825 |
| ApgarScore1min | 0.2480 |  |  |
| Totalattachment | 0.7313 |  |  |
| FetusGender | 0.6878 |  |  |
| PUQE | 0.6700 |  |  |

While the Apgar Score at 5 min turned out borderline significant, we did not consider this to be a statistically reliable effect: Firstly, for three out of the nine babies the measurement was missing altogether, and for all other, except two babies, this score had the value 9. For the mentioned two babies, the Apgar5min score took the values 8 and 10. Hence, these two babies have a strong `leverage effect’ in the sense that their values on the vertical axis essentially determine the slope of the effect. Hence, we do not consider this a genuine effect. The situation is also visualized in the following figure; each colour corresponding to a specific baby.


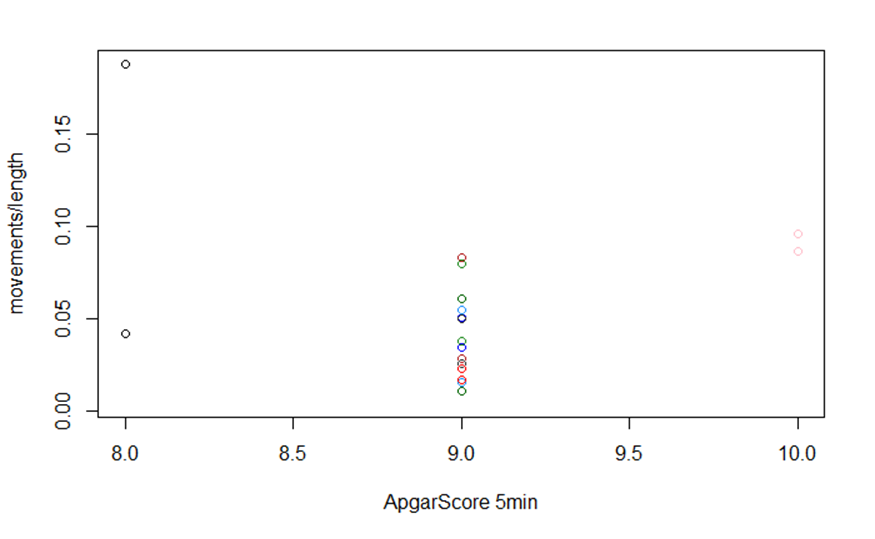


**Supplementary information S4: Coefficient estimates for model (C)**

Italic rows correspond to the model without the score variables.

| Coefficient | Estimate | S.E. | z-value | p-value |
| --- | --- | --- | --- | --- |
| Intercept | -3.8702  *-3.3603* | 0.3423  *0.2429* | -11.305  *-13.836* | <2e-16  *<2e-16* |
| catabolism | 0.6692  *0.9176* | 0.3922  *0.4089* | 1.706  *2.244* | 0.088  *0.025* |
| GestationalAge | 0.2421  *0.1757* | 0.3110  *0.3407* | 0.779  *0.516* | 0.436  *0.606* |
| catabolism:GestationalAge | -0.5788  *-0.8392* | 0.5632  *0.5836* | -1.028  *-1.438* | 0.304  *0.150* |
| PSS | 0.0734 | 0.0426 | 1.723 | 0.085 |
| HADS_A | 0.0365 | 0.0479 | 0.762 | 0.446 |
| HADS_D | -0.1335 | 0.0719 | -1.857 | 0.063 |

**Supplementary information S5: Interaction plots for distinct movements**

We provide interaction plots for all ten distinct movement types. The horizontal axis corresponds in all panels to gestational age [weeks], and the vertical axis to the mean number of movements per codable scan length, of the respective moment type. The scan length is measured in seconds [s], so the vertical axis has unit 1/s. In all plots, dashed lines correspond to the relative number of movements for healthy mothers, and solid lines under HG. These plots can be compared to Figure 2a from the main manuscript, which provides the equivalent information for the total number of movements.


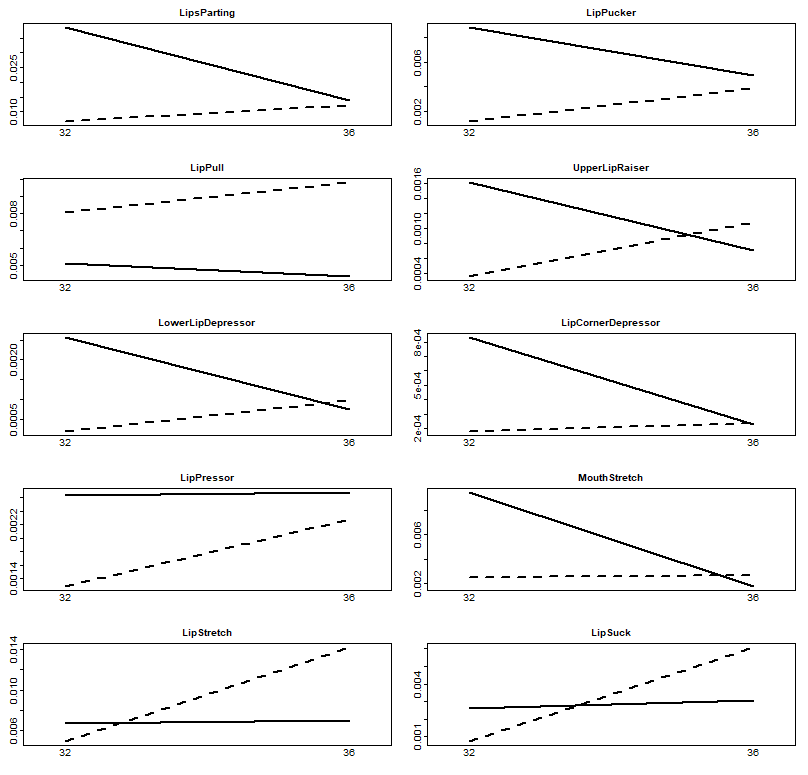

Supplement: Supplementary file 1 — Supplementary file1 (DOCX 230 kb) [file 404_2020_5571_MOESM1_ESM.docx]
